# The Development of a WTC Environmental Health Center Pan-Cancer Database

**DOI:** 10.3390/ijerph18041646

**Published:** 2021-02-09

**Authors:** Yongzhao Shao, Nedim Durmus, Yian Zhang, Sultan Pehlivan, Maria-Elena Fernandez-Beros, Lisette Umana, Rachel Corona, Adrienne Addessi, Sharon A. Abbott, Sheila Smyth-Giambanco, Alan A. Arslan, Joan Reibman

**Affiliations:** 1 Division of Biostatistics, Department of Population Health, NYU Grossman School of Medicine (NYUGSOM), New York University, New York, NY 10016, USA; yian.zhang@nyulangone.org (Y.Z.); alan.arslan@nyulangone.org (A.A.A.); 2 Department of Environmental Medicine, NYUG-SOM, New York University, New York, NY 10016, USA; 3 World Trade Center Environmental Health Center, NYC Health + Hospitals, New York, NY 10016, USA; nedim.durmus@nyulangone.org (N.D.); sultan.pehlivan@nyulangone.org (S.P.); mariaelena.fernandez-beros@nyulangone.org (M.-E.F.-B.); lisette.umana@nychhc.org (L.U.); coronar@nychhc.org (R.C.); addessia1@nychhc.org (A.A.); sharon.abbott@nychhc.org (S.A.A.); sheila.smyth-giambanco@nychhc.org (S.S.-G.); 4 NYU Perlmutter Comprehensive Cancer Center, New York, NY 10016, USA; 5 Division of Pulmonary Medicine, School of Medicine (SOM), NYUG-SOM, New York University, New York, NY 10016, USA; 6 Department of Obstetrics and Gynecology, School of Medicine (SOM), NYUG-SOM, New York University, New York, NY 10016, USA

**Keywords:** cancer incidence, biomarkers, WTC Environmental Health Center, September 11th, clinical cancer database, WTC survivors

## Abstract

(1) *Background:* Recent studies have reported elevated risks of multiple cancers in the World Trade Center (WTC) affected community members (also called WTC “Survivors”). The large variety of WTC-cancers created a need to develop a comprehensive cancer database. This paper describes the development of a pan-cancer database at the WTC Environmental Health Center (EHC) Data Center. (2) *Methods:* A new REDCap-based pan-cancer database was created using the pathology reports and available biomarker data of confirmed cancer cases after review by a cancer epidemiologist, a pathologist, physicians and biostatisticians. (3) *Results:* The WTC EHC pan-cancer database contains cancer characteristics and emerging biomarker information for cancers of individuals enrolled in the WTC EHC and diagnosed after 11 September 2001 and up to 31 December 2019 obtained from WTC EHC clinical records, pathological reports and state cancer registries. As of 31 December 2019, the database included 3440 cancer cases with cancer characteristics and biomarker information. (4) *Conclusions:* This evolving database represents an important resource for the scientific community facilitating future research about the etiology, heterogeneity, characteristics and outcomes of cancers and comorbid mental health conditions, cancer economics and gene–environment interaction in the unique population of WTC survivors.

## 1. Introduction

The destruction of the World Trade Center (WTC) towers on 11 September 2001, released a vast amount of pulverized material into the surrounding environment [1,2,3]. The dust settled on the streets and buildings in the local community of the WTC site and breached the indoor areas, necessitating a massive and prolonged clean-up. Fires from the combustion of the collapsed towers continued through December 2001. Community members, including those who lived, worked, attended schools or were involved in cleaning many of the surrounding areas affected by the WTC exposure, now also called “WTC Survivors”, were at risk of massive acute exposure from the dust clouds from the collapsing buildings on 9/11 or chronic exposure from resuspended dust and fumes from the fires.

WTC survivors with certifiable physical and mental health conditions are enrolled in the WTC Environmental Health Center (EHC), a federally designated treatment and surveillance program for WTC survivors. Extensive clinical and demographic information and WTC exposure status are obtained during the evaluation phases at WTC EHC and included in a clinical database at the WTC EHC Data Center. Approximately 50% of the members of the WTC EHC report dust cloud exposure on 11 September 2001 [4], and those and many others were also exposed to the subsequent resuspended dust and fumes. Local workers and residents began returning to the WTC site and local area approximately one week after the event, and were at risk of chronic exposure from resuspended dust from ongoing clean-up activities, incompletely remediated spaces, contaminated ventilation systems, self-cleaning activities and fumes from ongoing fires. Many local residents reported physical damage to their homes, with high rates of indoor WTC dust and fumes. Many children have been exposed to WTC dust at home [2,5,6]. We and others showed the onset of clinical symptoms and a dose–response relationship between dust/odors in the home and onset of adverse respiratory symptoms and signs in community members [7,8,9,10].

The WTC dust and fumes contained known carcinogenic compounds (e.g., asbestos, metals, polyaromatic hydrocarbons and dioxins). Soon after the terrorist attack, there were concerns about potentially elevated cancer risk among WTC survivors and Responders exposed to a complex mixture of toxic chemicals [2,11,12,13]. The James Zadroga 9/11 Health and Compensation Act of 2010 (H.R. 847) was passed by the US Congress, as a law designed to respond to the adverse health effects of the disaster, and was authorized in December 2010 and reauthorized in 2015. This act coordinated the clinical and surveillance programs that had been initiated by community groups and included them under the umbrella WTC Health Program (WTCHP) under Centers for Disease Control and Prevention—National Institute for Occupational Safety and Health (CDC-NIOSH) [4]. The WTCHP created inclusion criteria for enrollment for treatment in this program, which required defined WTC exposures, and for community members only, the presence of a certifiable WTC-related health condition [4]. We previously showed that WTC acute and chronic exposures are significantly associated with lower respiratory symptoms (LRS), lung function abnormalities and neuropathic symptoms [14,15,16,17,18]. Moreover, our documentation of psychological exposures and relevant mental health symptoms (PTSD, anxiety and depression) allowed longitudinal mental health analysis and we showed that WTC dust exposures are associated with mental health disorders [19,20] as well as physical health symptoms of WTC survivors, including both adults and children [4,5,21,22,23,24,25,26,27].

As increased cancer rates began to be described in WTC responders and WTC survivors [28,29,30], many cancers were added to the list of WTC-related and certifiable conditions in 2012 (https://www.cdc.gov/wtc/conditions.html (accessed on 28 June 2020)). Now, more than 19 years after the WTC terrorist attack, the WTC environmental exposure is becoming etiologically relevant for cancer, and the aging cohort is entering a period in life when cancer becomes more frequent, making the effective conduct of cancer research in both WTC responders and WTC survivors critical and feasible.

Most published information on WTC cancer rates and types is derived from the screening programs of the WTC Responder populations. An early case series in WTC responders reported multiple myeloma (MM) with cases noted in individuals less than 45 years old [29]. Increased prostate and thyroid cancer rates have been reported in three cohorts of the WTC responders [28,31,32,33,34]. Importantly, studies that included non-Responders performed by the WTC Health Registry (WTCHR) suggest an increase in all-cancer standardized incident rates [35] with breast cancer and non-Hodgkin’s lymphoma significantly elevated in WTC survivors [35]. The rapid increase in the number of Survivors with cancers makes it imperative to characterize these cancers in detail for efficient cancer management.

The WTC Environmental Health Center (WTC EHC) was included as the only “Center of Excellence” for the treatment of affected community members, now commonly referred to as “WTC Survivors,” in the WTCHP designated by CDC/NIOSH. A subsequent national program was developed, which includes some “Survivors”. By law, enrollment in the WTC EHC required defined WTC exposure, as well as the presence of a certifiable WTC-related health condition [4]. Enrollment remains open to the community members. Information about patients in the WTC EHC is included in a clinical database that includes extensive clinical information, which is obtained during the initial evaluation and subsequent monitoring phases. This information allows for detailed descriptions of patient characteristics as well as WTC and other environmental exposures. In contrast to the responder programs, the WTC EHC includes patients with diverse race/ethnicity and income, and nearly 50% are women [36]. The diverse social-economic statuses (SES) of the WTC EHC patients allow the study of a potential association between low income and cancer diagnosis and other health disparities. Since cancers were added as WTC-related health conditions in September 2012, more than 3000 cancer diagnoses have been identified in the WTC EHC clinics. The number of WTC EHC enrollees with WTC-cancers is still increasing.

The reauthorization of the James Zadroga 9/11 Health and Compensation Act affords a valuable opportunity to study cancers described among those exposed to the WTC dust and fumes. The diverse population in the WTC, including the large number of women, provides an exposed population that differs from the WTC responders. Importantly, the large variety of cancers described in WTC-exposed populations created an urgent need to develop a comprehensive cancer database that allows for information to be gathered on all WTC cancers. To address this need, we created a WTC EHC pan-cancer (Pan-Can) database that includes available cancer diagnoses and captures comprehensive cancer and available biomarker information and biopsied tissues on all cancer types, as well as the presence of consent to re-contact patients to collect more patient-specific information, such as family cancer history. This information can be linked to the WTC EHC clinical database that contains information on mental and physical comorbidities, as well as information about WTC and other exposures (e.g., smoking and alcohol consumption) and demographic information.

We now present information about the structure of the database as well as summary data to illustrate the properties of the WTC EHC pan-cancer database. This database will allow investigations about individual cancer types, common characteristics across WTC-related cancer types and support subsequent translational research and epidemiologic follow-up studies. We describe the creation and components of this database, with the intent to facilitate future study of relationships between WTC exposure and cancers, cancer heterogeneity, cancer characteristics and associated comorbidities. Additionally, given WTC EHC is a federally designated treatment program for interdisciplinary illnesses in WTC survivors, the database can promote the study of how the mental and physical comorbidities relate to WTC and how other exposures affect cancer risk and/or cancer latency after 11 September 2001, as well as cancer outcomes. In addition, the database can undergo future longitudinal expansion for use in evaluating the effectiveness of cancer therapies and follow-up of cancer patients. We now report on the development and structure of the WTC EHC pan-cancer database for the cancers identified in the WTC EHC after 11 September 2001 and up to 31 December 2019.

## 2. Methods

### 2.1. Dataset Development

The development of a pan-cancer multidisciplinary database with intent for future expansion is a challenging endeavor for several reasons. The appropriate data fields have to be carefully defined and created with the intent for future epidemiological and translational research of various cancer conditions while minimizing important data gaps and missing information and allowing for emerging information. In addition, the collection and archiving of extensive clinical data including information on cancer characteristics for many types of cancers, patient demographics, key biomarker data and follow-up information have to be carefully annotated and validated. The pan-cancer database was, therefore, developed with the collaboration of an interdisciplinary group of clinicians representing a variety of clinical disciplines, epidemiologists and biostatisticians, as well as a dedicated staff for document and data retrieval.

### 2.2. The HIPPA-Compliant REDCap Pan-Cancer Database

The storage of data in a secure database is also of the utmost importance. We adopted the Research Electronic Data Capture (REDCap) system as a secure FISMA and HIPAA-compliant environment, which is geared to support online and/or offline data capture for research studies (https://www.project-redcap.org (accessed on 28 June 2020)). REDCap has a secure web application that manages online databases and surveys and is widely used by many academic institutions allowing for easy accessibility. It can be conveniently utilized to provide the integration layer to the de-identified clinical and laboratory data with the help of various application programming interfaces (API) that can transfer the data into the system. REDCap’s flexibility and its foundation in MySQL allow it to store the information in various relational database tables that are easily implemented, making it an ideal choice for this type of data collection. The dynamic data pull (DDP) feature of REDCap can be utilized to provide data interoperability between various data sources to pull information directly from various systems providing the raw data for various translational research projects. In addition, DDP’s push feature can be conveniently used to provide real-time data dashboard access through Tableau, a widely used analytic platform that provides basic analytics and can be used as an easy way of visualizing data.

### 2.3. Development of the WTC EHC Pan-Cancer Database

Overall, the database includes information on patient demographics, cancer characteristics and types and cancer biomarkers. The development of these main components of the database is described in the next subsections.

#### 2.3.1. Identification of WTC Exposure and Demographic/Clinical Information in the WTC EHC

The WTC EHC pan-cancer database was developed to be merged easily with the WTC clinical database at the WTC EHC. The WTC EHC clinical database includes information on WTC and other exposures (e.g., smoking); demographics; physical and mental health symptoms, including information on time of onset and symptom severity; laboratory values, including basic blood studies; and measures of lung function [4] and insurance claims data. Importantly, information on whether the patient has signed consent to have their information used for research, as well as whether they agree to be re-contacted, is maintained in this database. Information is obtained at initial evaluation and subsequent monitoring visits. Information about death is obtained from patient family reports or the National Death Index. The WTC EHC clinical database includes information on patients with and without cancers and is maintained at the WTC EHC Data Center approved by the New York University IRB.

WTC exposure information is captured in the WTC EHC clinical database with numerous queries about location WTC exposures, types of exposures and duration of exposures. Due to the complexity of WTC exposures in a diverse population, we routinely classify acute exposure as exposure to WTC dust clouds on 11 September 2001 and chronic exposure as exposure to WTC re-suspended dust and fumes post 11 September 2001. Patients are at risk for both types of exposure. In addition, we classify patients by their potential for WTC exposure as clean-up worker, resident, student, local worker and other. This detailed information on WTC and other exposures (e.g., smoking) are captured in the initial visit questionnaires at WTC EHC, allowing for more detailed WTC exposure analyses in the future.

Detailed information is captured on type, onset and severity of numerous physical symptoms. We previously used the WTC EHC clinical database to conduct and publish research on lower respiratory symptoms (LRS) and neuropathic symptoms [4,14,15,16,17,18,36]. In addition to exposures to WTC dust and fume, the WTC survivors were also exposed to traumatic psychological exposures, and extensive data have been collected on mental health symptoms of PTSD, depression, anxiety and substance use [19].

#### 2.3.2. Ascertaining Cancer Types and Characteristics in the WTC EHC Pan-Cancer Database

To facilitate understanding of the relationship between environmental exposures and cancers, our WTC EHC pan-cancer database was developed to include detailed information on cancer characteristics. This information is obtained from a careful review of pathology reports, medical records or state tumor registries with documentation of sources included. Information from pathology is reviewed by a pathologist for appropriate interpretation and input of the data. Cancers are identified from self-reports as currently enrolled patients that require support for treatment, newly self-referred patients who are enrolling in the program, as well as from linkages with state cancer registries.

#### 2.3.3. Identification of Cancer Biomarker Information

The WTC EHC pan-cancer database has the potential to include information on approximately 1000 cancer-related biomarkers. These potential biomarkers were identified after careful review of the literature, medical records, pathology reports and documentations from state cancer registries. Biomarkers include protein markers, lipid markers and genetic markers. Multiple markers can be included for each cancer, and can be updated as appropriate. The biomarker, date of accession, laboratory, result, including quantified result and result interpretation are all included, when available. Biomarker results are included from medical and pathology reports and addendum pathology reports of biopsies and surgical procedures of cancer patients. Data input is reviewed by a clinician and a pathologist to resolve any ambiguities.

#### 2.3.4. Ethical Statement

The database was created under the approval of the New York University School of Medicine Institutional Review Board (IRB). All data from this database will be used for further studies according to the ethics statements in the approval (IRB number: i06-1). If any reference group of non-cancer participants is used, the participants who have signed consent for analysis of their data will be included in the study. Patients with cancer will be analyzed after removal of personal identifiers with IRB approval to review de-identified data (IRB number: i06-1_MOD49). Documentation of consent to be re-contacted is included for subsequent studies.

## 3. Results

### 3.1. Structure of the WTC EHC Pan-Cancer Database

**Figure 1:**
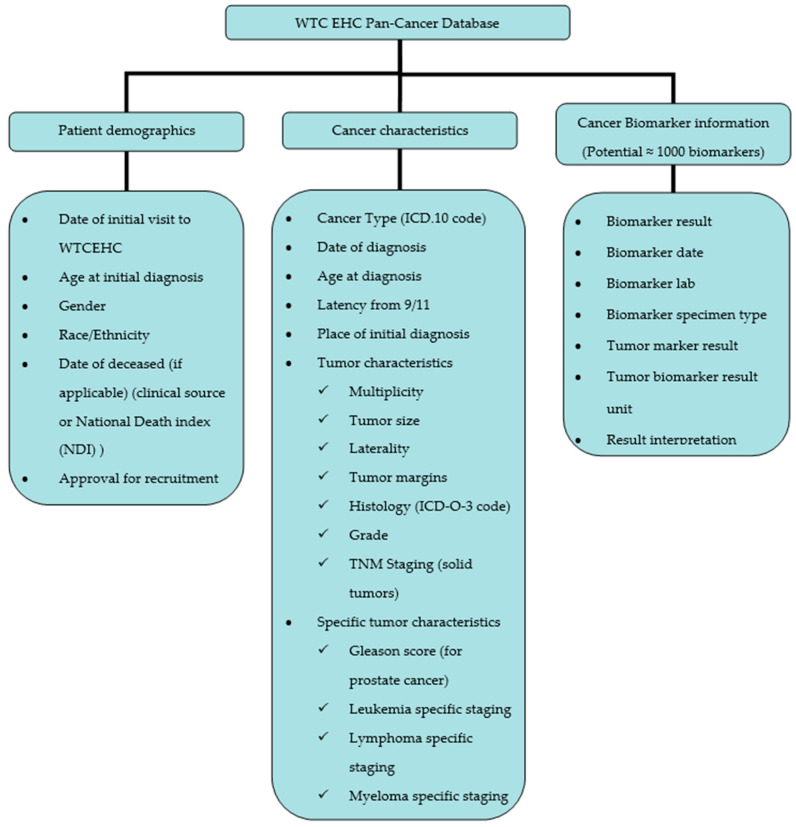
WTC EHC pan-cancer database overview.

The overall structure of the WTC EHC pan-cancer database is shown in Figure 1. Briefly, the database includes information on patient demographics, cancer characteristics and cancer biomarkers. Examples of the appearance and user interface of the WTC EHC pan-cancer database in REDCap are shown with a screenshot of the blank database fields in the supplemental material (Figure S1).

### 3.2. Inventory

#### 3.2.1. Demographic Information in the WTC EHC

The WTC EHC pan-cancer database can be linked to the WTC EHC clinical database which has clinical and demographic information on all WTC EHC enrollees including those who have not yet developed any cancers, allowing for additional studies. An example of this combined information is shown in Figure 1, for all patients enrolled in the WTC EHC up to 31 December 2019 with any “certifiable cancer”. To date, our pan-cancer database contains 3440 primary cancer cases from 2840 patients. Based on merged data from the pan-cancer database and the clinical database, Table 1 presents some of the key demographic characteristics of the cancer patients in the WTC EHC pan-cancer database. In general, of these cancer patients, the median age on 9/11 was about 46.5 years, and about half of subjects were females. The majority of subjects were overweight, had an income higher than USD 30,000 per year and an education level above high school. More than 50% reported having been caught in the WTC dust cloud on 11 September 2001. The majority are as local workers, with fewer residents or other categories. One primary cancer was reported in 2382 patients (83.9%), 355 (12.5%) had two primary cancers and 103 (3.6%) had records of three or more primary cancers. The database also contains three patients who had their first primary cancer diagnoses under 18 years of age (mean = 12 years old, SD = 4.6). Two of them are male, and one is female with leukemia, thyroid and kidney cancers.

**Figure 2:**
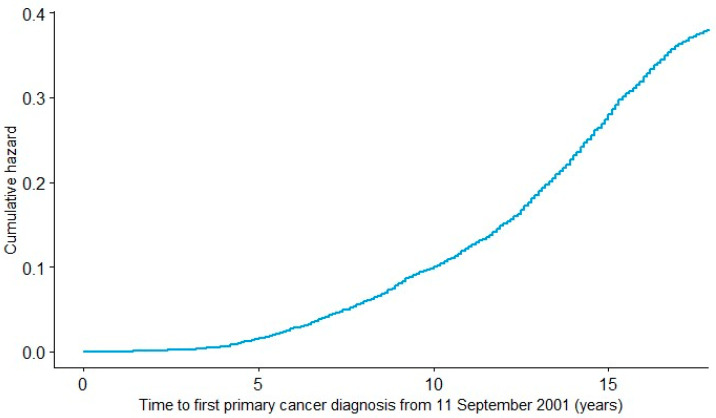
Cumulative hazards for first primary cancer diagnosis from 11 September 2001 among cancer patients in WTC EHC pan-cancer database.

The pan-cancer database can be linked to the WTC EHC clinical database which has clinical and demographic information on all WTC EHC enrollees, including those who have not yet developed any cancers. This information can facilitate future translational and clinical studies. For example, the cumulative hazards for the first primary cancer diagnosis from 11 September 2001 among cancer patients up to 31 December 2019 are shown in Figure 2. Clearly, Figure 2 indicates that the hazard of first primary cancer diagnosis has increased dramatically in recent years and the trend is still increasing. This indicates the need and urgency to conduct research on WTC cancers in this population and the need to develop a cancer database to facilitate research. This increasing trend of the hazard function in Figure 2 can be due to many factors, including that the WTC environmental exposure is becoming etiologically relevant for cancer as discussed in the literature, the aging cohort is entering a period in life when cancer becomes more frequent and the potential for accumulation of sufficient genomic alterations conferring growth and survival advantages of transformed neoplastic cells, amongst other factors, making the development of this pan-cancer database a valuable tool to facilitate future effective conduct of cancer research among WTC survivors.

#### 3.2.2. Characteristics and Cancer Types in the WTC EHC Pan-Cancer Database

According to the NIOSH guidelines and standards, patients enrolled in the WTC EHC are required to have defined “certifiable” physical and/or mental health conditions. Many cancers are included under the NIOSH guideline, including non-melanoma skin cancers. A few selected cancers that affect women, such as cervical or uterine, have been excluded according to federal rules. As illustrated in Figure 1, the cancer type, date and age at diagnosis, ICD.10 description/code, laterality, location of initial pathology report, multiplicity (number of tumors at primary site), tumor margins, grade, tumor histology (ICD-O-3 code), TNM classification of tumor, stage and tumor size are all included. Importantly, the medical location where the biopsy was performed is included in order to enable tumor sample accession if desired. Standard tumor-specific information is noted when appropriate and includes the Gleason score for prostate cancer, Rai staging system, Binet Classification, FAB classification for leukemias, Ann Arbor classification—Lugano classification for lymphomas, International Staging System and Durie-Salmon System for multiple myeloma. In short, our database contains clinical, pathologic and key biomarker information of cancers at the WTC EHC. Detailed descriptive characteristics of the cancers from this pan-cancer database were discussed by Durmus et al. [37].

**Figure 3:**
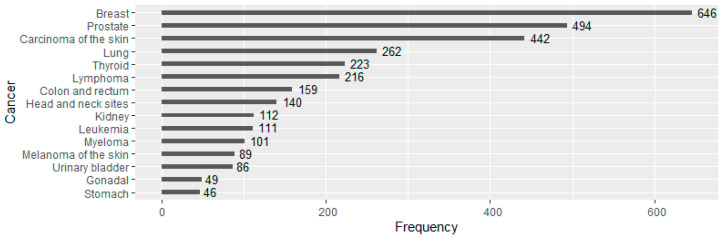
Top 15 cancer diagnoses in WTC EHC pan-cancer database.

The top 15 cancer diagnoses, including non-melanoma skin cancers, are presented in Figure 3. Top-ranked cancers stratified by gender are summarized in Table 2. Breast cancer was the most prevalent cancer in the WTC EHC total population (*n* = 646 cases) as well as in the female subgroup (*n* = 635). About 42% of the female cancer patients had breast cancers, followed by lung (10%), carcinoma of the skin (i.e., non-melanoma skin cancer, 9%), thyroid (8%) and lymphoma (6%). Prostate cancer was the most frequent cancer among males (*n* = 494), which was diagnosed in 26% of the male cancer patients, followed by carcinoma of the skin (16%), lymphoma (7%), lung (6%) and head and neck sites (6%). In addition, there were 11 breast cancer cases among male WTC survivors.

#### 3.2.3. Cancer Biomarker Information

The WTC EHC pan-cancer database has a potential to include up to 1000 cancer-related biomarkers that were identified after comprehensive review of the cancer literature. These biomarkers include protein, lipid and genetic markers. For each cancer diagnosis, we carefully review all available medical records, pathology reports and documentation from state cancer registries. Multiple markers can be entered into the database for each cancer. For example, for breast cancer, we collect the status of standard biomarkers, estrogen receptor (ER), progesterone receptor (PR), human epidermal growth factor receptor 2 (HER2) receptor and BRCA mutation status, when available. For lung cancer, we collect the status of the National Comprehensive Cancer Network (NCCN) recommended biomarkers: EGFR, ALK, BRAF, ROS1, KRAS, RET, NTRK and PD-L1. Biomarkers are not restricted for the type of cancer. As biomarkers are identified to be clinically important, we can add these biomarkers to each cancer. We include the name of the biomarker, date of accession, name of laboratory and laboratory result (including quantified result, unit and result interpretation). Data input is jointly reviewed by a clinician and a pathologist to resolve any ambiguities.

## 4. Resource Dissemination

### 4.1. Establishing the WTC EHC Pan-Cancer Database as a Resource for WTC-Related Scientific Community

The WTC EHC pan-cancer database complements data from the WTC Responder programs, the Mount Sinai BioBank for WTC responders [30], the New York City Department of Health WTC Health Registry (WTCHR) and information derived from the Fire Department of New York (FDNY). Importantly, the FDNY and other WTC Responder programs include predominantly white adult males who may not accurately represent the cancer incidence and comorbidity in disaster-affected general populations. In contrast, the different exposures documented for the WTC survivors, the diverse race/ethnicity and gender distribution in this population compared with other WTC cohorts make it imperative to study this civilian cohort. About half of the WTC EHC population are women. This provides a unique opportunity to study cancers affecting women, who may be impacted differently by the WTC exposures compared to men.

The WTC EHC also enrolled affected young adults and children, and we previously published on the health conditions of the WTC-affected children and relationships between their WTC exposures and children’s physical and psychological outcomes [5,24,25,26,27,38,39]. Since enrollment into the WTC EHC remains open, patients who develop cancers will continue to enroll in the program and will be included in the WTC EHC pan-cancer database. This will enable a detailed evaluation of cancers in this population who were exposed as children.

The WTC EHC pan-cancer database is a valuable resource to study the interplay of environmental exposure, comorbid medical and mental disorders and cancers. In addition to exposures to WTC dust, gas and fumes, many WTC survivors were also exposed to traumatic psychological exposures. We collected data on mental health outcomes, including information on PTSD, anxiety and depression, in our WTC EHC clinical database. As is well known, cancer diagnosis causes excess mortality in those with mental disorders in the United States [40,41,42]. On the other hand, extensive literature points to the evidence that mental disorders are a risk factor for incident cancer, including breast cancer, with high population attributable risk [43,44,45]. We and others have used the exposure data, physical and mental health outcomes for studies characterizing respiratory, mental health, neurologic and sleep symptoms in the WTC EHC enrollees [14,17,19,46,47,48,49,50,51,52]. The new WTC EHC pan-cancer database can be linked with our clinical database for improved characterization of WTC and other exposures and demographics of individual cancers, comparison across cancers, as well as studying mental health disorders and other conditions as potential mediation and moderation factors for cancer risks and/or cancer outcomes.

The WTC EHC pan-cancer database is a useful resource to design new translational studies, check patient availability and study feasibility. Importantly, the WTC survivors share, to a varying degree, WTC-related environmental exposures, including a mixture of dust, fume and chemicals, and psychological stress, including PTSD, depression and anxiety. Thus, it makes sense to study multiple cancers together to borrow strength on potential detectable relationships between shared environmental exposure and the WTC certified cancers. The WTC EHC pan-cancer database facilitates the study of WTC environmental exposure impact. The impact on one particular cancer type might be small, but the effect or impact on multiple cancers might be easier to detect and investigate; thus, the pan-cancer database is useful in this aspect. For example, DNA copy number alterations (CNAs) can underlie multiple different cancers. We previously reported a pan-cancer CNA-based signature that was underlying the risk of breast cancer, melanoma and multiple cancers [53]. In addition, the WTC EHC pan-cancer database is also useful for future studies of persons with multiple primary cancers or rare cancers.

### 4.2. Dissemination

The WTC EHC pan-cancer database will be presented to the scientific community through posters at scientific meetings and cited in articles for publication in medical and environmental science literature. Outreach and advertising of the WTC EHC pan-cancer database will also be accomplished with WTCHP, WTC responder programs, WTC survivors steering committees, WTC research communities and New York University Perlmutter Comprehensive Cancer Center (PCCC) and the New York Genome Center. The existence of the database and the results generated from the database will be communicated to participants of semi-annual WTC research meetings, as well as participants in other WTC surveillance programs. We collaborated with the NCI designated NYU Perlmutter Comprehensive Cancer Center and their community outreach program to encourage collaborations between NYU PCCC and WTC health researchers using this database as a stepping stone. A major objective of developing this database is the potential availability of the de-identified data to the scientific community, aimed at facilitating high impact research on WTC environmental exposures and disease etiology, cancer survival and biomarker-environment interactions in the WTC affected community, including both WTC responders and WTC survivors.

We established a process for qualified applicants to request available data for use in research projects using the WTC EHC pan-cancer database. We also formed the Database Utilization and Coordinating Committee (DUCC) and a Research Evaluation Panel (REP). The REP consists of the WTC EHC Medical Director, the Principal Investigator of the WTC EHC pan-cancer DB, a cancer epidemiologist, a biostatistician, a pathologist and other members of the cancer research community and is responsible for determining the priority and importance of the proposed studies, the area of weakness of study design that requires improvement. The REP will summarize the review in a written report that will be communicated to DUCC and maintain records of these processes. The DUCC oversees and guides the development and utilization of the WTC EHC pan-cancer database. The DUCC prepares the Manual of Operations for establishing uniform procedures to access, process and distribute de-identified data. After final approval by the DUCC is granted, the research institution requiring the data must provide IRB approval or a waiver letter for the project, and a data use agreement between the institution and the WTC EHC pan-cancer database must be created. This ensures that patient data are only being used for their specific and approved purposes. The WTC EHC will share only anonymized data, ensuring there is no link to participants.

Among many attractive functions, including being a secure FISMA- and HIPAA-compliant environment, REDCap’s capability of remotely capturing online surveys is an attractive feature for conducting online surveys, which is particularly important in the post-COVID-19 pandemic era and for many telemedicine applications. Although there are numerous REDCap databases for a specific disease or a specific cancer type in many institutions already, to the best of our knowledge, our WTC EHC pan-cancer database is the first REDCap database for pan-cancers. In addition to sharing anonymized datasets, we are also ready to share the template of our REDCap pan-cancer database so other researchers may adapt or adopt our database template and similarly develop their own multidisease or multicancer database sharing a common exposure or a common treatment.

## 5. Discussion

This WTC EHC pan-cancer database will facilitate cancer studies of WTC survivors and will complement the cancer studies on WTC responders. There have been many published cancer studies for a variety of cancers mostly in WTC responders [28,29,30]. There are many important reasons for the development of a database that captures comprehensive information on all the cancers in this environmental disaster-exposed population. Early data suggest the development of multiple cancer types (hematologic, prostate and thyroid) in responder populations. This finding raises the question of whether WTC exposures altered underlying mechanisms of cancer development affecting cancer suppressor genes and oncogenes. The WTC EHC pan-cancer database focuses on all WTC cancers rather than one type of cancer. The inclusion of all cancer types, and collection of data on a large number of biomarkers, may facilitate epidemiologic studies on cancer molecular characteristics as well as cancer subtypes that are more common in the WTC-exposed populations. Thus, our WTC EHC pan-cancer database facilitates the study of cancer biomarkers and allows for a more complete understanding of the relationship between WTC exposures and molecular and functional pathways involved in cancers. Our use of a widely accepted database structure (REDCap) facilitates sharing the database templates and anonymized datasets with collaborators and researchers at other institutions. This will become even more important as we expand upon the advanced functions and tools that the REDCap system can offer, including longitudinal data collection and follow-up of cancer patients.

The capacity to study multiple cancers facilitates the borrowing of strength across cancers and increases statistical power for detecting the effect of environmental exposure on WTC cancers. Furthermore, for statistical analysis, it is well known that the onset of multiple possible cancers is a competing time-to-event variable. Investigating risk factors on only one cancer per study essentially ignores the competing risk of other cancers and other disorders, which violates important assumptions of the commonly used statistical models and can lead to biased conclusions. On the other hand, possible pathophysiologic mechanisms leading to multiple cancers and mostly accompanying cancers with each other could not be investigated without a pan-cancer database. The WTC EHC pan-cancer database facilitates the study of multiple cancers and other physical and mental health conditions after WTC environmental exposure that can potentially reduce the biases of ignoring competing risks of single cancer studies while providing increased statistical power to detect associations between environmental exposure and WTC-related cancers. Additionally, detailed information on biomarkers may help suggest possible connections and mechanisms between environmental exposures and multiple developed cancers.

This WTC EHC pan-cancer database will facilitate studies of WTC cancers in diverse affected civilian populations and will complement the cancer studies on WTC responders. Whereas WTC responders are predominately white adult males, the WTC EHC contains about 50% women, making the WTC EHC pan-cancer database a valuable resource to gain in-depth understanding on how the WTC exposures impact women’s health. Our WTC EHC pan-cancer database also facilitates the investigation of health disparity and evaluation of the overall health impact of WTC environmental exposure in the context of an affected community of civilian population with diverse socioeconomic status and racial/ethnic background.

The WTC EHC pan-cancer database expands our ability to study environmental and other exposures in this community population. The WTC EHC pan-cancer database can be merged with databases that include both acute and chronic WTC exposure data as well as other exposure information, such as smoking, that can modify cancer risk. In addition, the WTC exposure categories in our data, including local workers, those involved in cleaning the surrounding area and local residents, can be useful to explore possible occupational and environmental sources of exposure in addition to dust and fume exposures due to the WTC terrorist attacks. The ability to analyze information on multiple WTC as well as other environmental exposures with information on multiple cancer types, cancer characteristics and cancer biomarkers has the potential to fully inform our understanding of the interplay of environmental exposures and cancer risk and mechanism. In particular, genomic and epigenetic research can be used to study the effect of WTC-related exposures on clinical cancer characteristics, and possible gene–environment interactions [53,54,55,56]. Establishing a link between occupation and duration and type of exposure to the substances released would be useful. Our questionnaires include complex questions on duration and timing of exposures. However, data on exposure to specific contaminants (e.g., asbestos) are generally not available for community members, which is a major limitation to the study of cancers in this WTC cohort and others.

The WTC EHC pan-cancer database is a unique resource to study the interplays of environmental exposure and comorbidities, including psychological stress, depression and anxiety and cancers in a civilian population that includes women and children. Examples of these studies that can be performed in the future include those involving SES, elevated BMI, as well as race/ethnicity and sex and mental health. As is well known, cancer diagnosis causes excess mortality in those with mental illness in the United States [40,41,42]. Depression is also a risk factor for incident cancer in community-based populations of adults with both a lifetime history of DSM-III major depression as well as of dysphoria and risk of overall cancer [43]. Persistent depression appeared to increase the hazard for cancer, particularly breast cancer among women [44,45], and among older persons based on a community-based prospective follow-up cohort study [57,58,59]. The population attributable risk of depression is likely sizeable and worthy of investigation given the considerable prevalence of depression symptoms in the WTC Survivor population. The ability to combine information on co-morbidities with information on multiple cancer types, cancer characteristics and cancer biomarkers has the potential to fully inform our understanding of these interrelationships.

There are several limitations in our current cancer database, some of which are outlined in our database description. Ideally, there is a need to supplement current databases with a cancer tissue bank and/or blood bank in order to conduct association study to elucidate the possible connection between characteristics of each cancer and WTC exposures. There are existing biobanks for WTC responders [30], but there is currently no blood bank or biobank for WTC survivors. However, the WTC EHC is a long-term surveillance program, and as such, through the WTC EHC pan-cancer database, we can identify patients who have agreed to be re-contacted, and have documentation of the location of biopsy specimens. The pan-cancer database will be maintained with the most up-to-date clinical information, including location of biopsy specimens. In addition, additional samples can be obtained as demonstrated by our previous study in which we showed that it is feasible to collect blood samples from current WTC EHC enrollees for translational studies [60].

Compared with national databases, such as the National Cancer Institute (NCI) Surveillance, Epidemiology and End Results (SEER) database and the Cancer Genome Atlas (TCGA) database, the WTC EHC pan-cancer database is a single center database. Therefore, caution is advised when generalizing results of a WTC EHC-based study to the broad patient population. In addition, there are some limitations of diagnoses, since patients enrolled in the WTC EHC have to have certifiable physical and/or mental health conditions according to NIOSH guidelines and standards. We do not have a control cohort, which would be important for future analyses. However, our database can be used in conjunction with other databases. Identification of suitable control cohorts is not straightforward, but finding control cohorts for specific cancers might be easier. An example of this is a pilot project we performed to study breast cancers in the WTC EHC where we used a population from the NYU Women’s Health Study, an existing prospective cohort with samples collected from 1995 and before 11 September 2001 as a reference group. Although many cancers are included, notably, selected cancers that affect women, such as cervical or uterine, have been excluded according to federal rules. Thus, there is a certain selection bias involved in the enrollment process. The database biomarker data are limited to those that are clinically requested for diagnosis or clinical management, and the clinical biomarkers obtained for clinical management with different cancers continue to change over time. Importantly, we included the potential for the involvement of up to 1000 biomarkers, allowing for expandable information as clinical biomarker data become more widely available.

Another limitation is due to limited resources for our clinical database. Professional quality pan-cancer databases require considerable resources to develop and maintain. The WTC EHC was initially the only federally designated treatment program for WTC survivors with the WTC EHC Data Center required to focus on billing and reimbursement of enrollees. Thus, the clinical data recorded were largely for clinical use with limited resources to support the manpower needed for data capture and formal extensive database development, and the valuable clinical data are maintained on a volunteer basis by our dedicated staff scientists for possible future research. Although this limited some scientific efforts, as a consequence, we have extensive data on billing and reimbursement, a valuable data resource linked to our pan-cancer database that facilitates future studies of health economics and the comparative effectiveness of cancer treatments.

Although incomplete, we are obtaining cancer data from the national program and other WTC programs, including close collaborations with WTC Health Registry (WTCHR) and WTCHP Responders Programs. Currently, the WTC EHC pan-cancer database only has cross-sectional data. We are in the process of expanding the database to capture longitudinal cancer follow-up data. In addition to providing useful clinical data, in the future, we could also help translational investigators to contact consented patients to obtain tumor tissues and blood for biomarkers and other translational research. The feasibility was demonstrated by a recent pilot research project on genome-wide DNA methylation profiles in community members exposed to the WTC disaster [60]. Indeed, the reauthorization of the James Zadroga 9/11 Health and Compensation Act affords an opportunity to study WTC cancers, including identifying new prognostic biomarkers for the better prediction of treatment outcomes, identifying new targets for cancer treatment and evaluating WTC cancer heterogeneity and comorbidity.

## 6. Conclusions

In summary, the WTC EHC pan-cancer database was developed for WTC survivors using the secure REDCap system. The WTC EHC pan-cancer database integrates extensive clinical and biomarker information on cancer with information on WTC and other exposures and combines information on medical and mental health comorbid conditions. It has a few unique features, including (1) allowing the ability to combine patient cancer clinical information with WTC exposure and additional non-WTC exposures (e.g., tobacco smoking, substance abuse and occupational exposures); (2) improving our analysis of cancer risk and environmental exposures; (3) enabling integration of cancer clinical information with other comorbidities, including mental health disorders to understand the interrelated joint outcomes or to understand how mental health comorbidity impacts cancer as a mediator or moderator; (4) facilitating investigation of cancers in a racially and ethnically diverse population; (5) increasing our ability to conduct longitudinal studies and other translational follow up studies for in-depth investigation of WTC cancers; (6) expanding our ability to understand not only an individual cancer but also multiple cancers in the context of WTC environmental exposure and competing risk setting; and (7) allowing for better understanding of cancer development and characteristics in WTC EHC in women as well as men. To date, it has compiled clinical and biomarker information for more than 3400 WTC-related cancer cases.

In conclusion, the WTC EHC pan-cancer database represents an important resource for the scientific community allowing for high impact studies on environmental exposures and cancer epidemiology, cancer etiology, cancer outcomes, comorbid mental health conditions and cancer economics in the unique population of WTC Survivors.

## Figures and Tables

**Table 1:** Demographic characteristics of cancer patients in the WTC EHC pan-cancer database.

Variable	Level	Cancer Patients
		*n* = 2840
Gender, *n* (%)	Female	1287 (45.3)
	Male	1553 (54.7)
Age on 9/11, median [IQR]		46.50 [39.50, 53.50]
Race/Ethnicity, *n* (%)	Hispanic	262 (11.3)
	Non-Hispanic White	1315 (56.8)
	Non-Hispanic Black	397 (17.2)
	Asian	337 (14.6)
	Native American	3 (0.1)
BMI, *n* (%)	Normal weight (<25)	541 (30.2)
	Overweight (25–30)	686 (38.3)
	Obese (≥30)	563 (31.5)
Income, *n* (%)	≤USD 30,000/year	926 (41.0)
	>USD 30,000/year	1335 (59.0)
Education, *n* (%)	High school or less	615 (26.1)
	More than high school	1743 (73.9)
WTC dust cloud, *n* (%)	Yes	1235 (52.4)
	No	1120 (47.6)
Exposure category, *n* (%)	Clean-up worker	19 (0.8)
	Resident	677 (28.8)
	Student	41 (1.7)
	Worker	1505 (64.1)
	Other	107 (4.6)
Number of primary cancers, *n* (%)	1	2382 (83.9)
	2	355 (12.5)
	3	74 (2.6)
	4	19 (0.7)
	5	10 (0.3)

**Table 2:** Top 15 cancer diagnoses in males and females in WTC EHC pan-cancer database.

Male			Female		
Cancer	*n* (1929 Cases)	%	Cancer	*n* (1511 Cases)	%
Prostate	494	26	Breast	635	42
Carcinoma of the skin	306	16	Lung	152	10
Lymphoma	130	7	Carcinoma of the skin	135	9
Lung	111	6	Thyroid	128	8
Head and neck sites	110	6	Lymphoma	87	6
Colon and rectum	101	5	Colon and rectum	58	4
Thyroid	95	5	Myeloma	43	3
Kidney	85	4	Head and neck sites	31	2
Leukemia	81	4	Leukemia	30	2
Urinary bladder	72	4	Ovary	29	2
Melanoma of the skin	65	3	Kidney	27	2
Myeloma	61	3	Melanoma of the skin	24	2
Stomach	31	2	Pancreas	16	1
Pancreas	28	1	Stomach	15	1
Esophagus	21	1	Urinary bladder	14	1
Other	138	7	Other	87	6

## Data Availability

The datasets in the WTC EHC pan-cancer database are not publicly available, but de-identified and anonymized information is potentially available upon reasonable request.

## Supplementary Materials

The following are available online at https://www.mdpi.com/1660-4601/18/4/1646/s1, Figure S1: The screenshot of the WTC EHC pan-cancer database in REDCap.

## Abbreviations

*CDC*	Center for Disease Control and Prevention
*DSM-III*	The Diagnostic and Statistical Manual of Mental Disorders, Third Edition
*FDNY*	Fire Department of New York City
*IOS*	impulse oscillometry system
*IRB*	Institutional Review Board
*IVQ*	Initial visit questionnaires
*LRS*	lower respiratory symptoms
*NCI*	National Cancer Institute
*NIOSH*	National Institute of Occupational Safety and Health
*PTSD*	post-traumatic stress disorder
*REDCap*	Research Electronic Data Capture web application for building/managing database
*ROC*	receiver operating characteristic curve
*SEER*	NCI Surveillance, Epidemiology, End Results database
*TCGA*	the Cancer Genome Atlas database
*WTC*	World Trade Center
*WTC EHC*	WTC Environmental Health Center
*WTCHP*	World Trade Center Health Program
*WTCHR*	World Trade Center Health Registry.
