# Supplementary material for: The Development of a WTC Environmental Health Center Pan-Cancer Database
Source: Int J Environ Res Public Health. 2021 Feb 9;18(4):1646. doi: 10.3390/ijerph18041646 (PMC7916067; doi:10.3390/ijerph18041646)
Supplement: Supplementary file 1 [file ijerph-18-01646-s001.pdf]

Figure S1: The screenshot of the WTC EHC Pan-cancer database in REDCap.

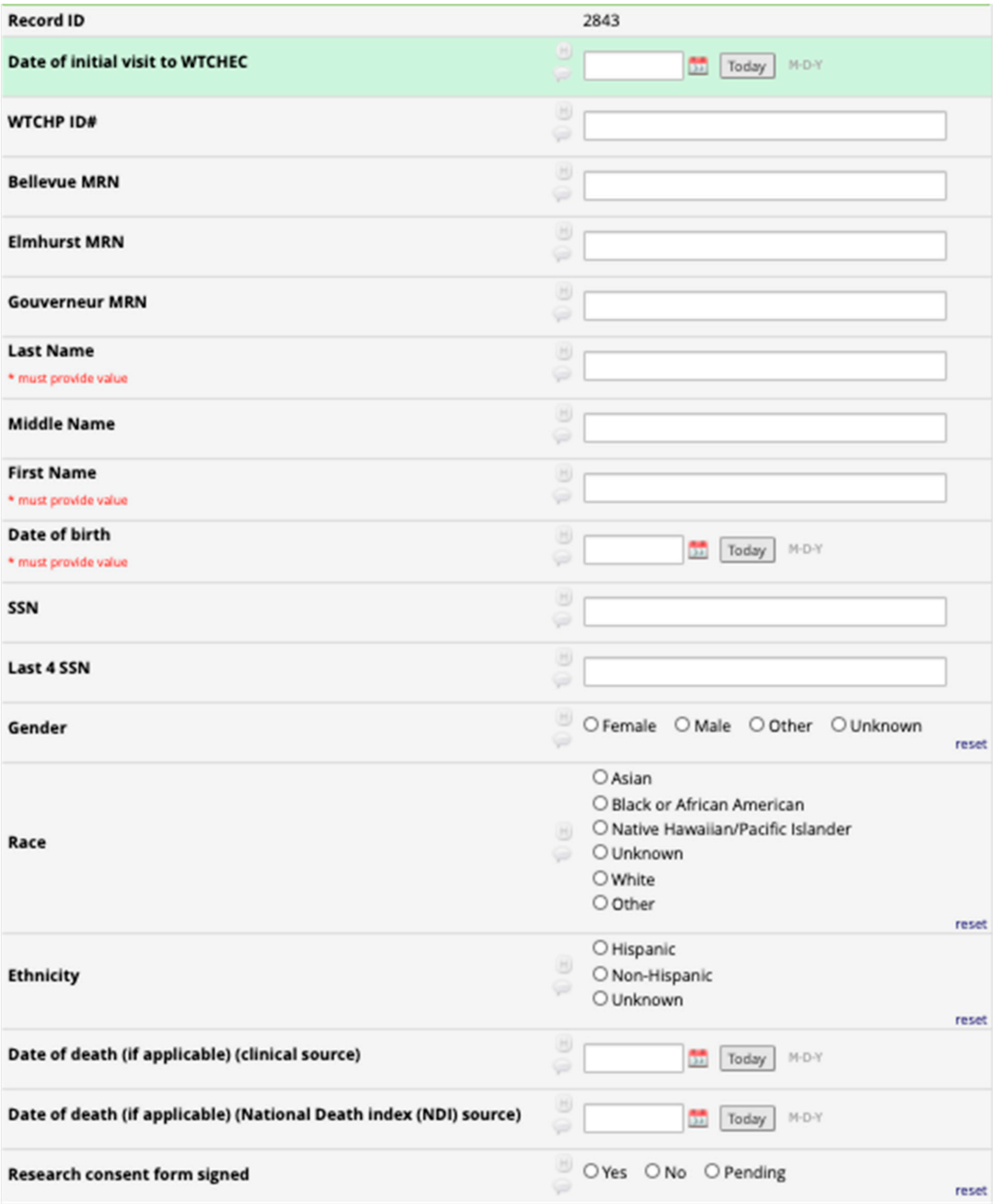

Record ID	2843
Date of initial visit to WTCHEC	Today M-D-Y
WTCHP ID#	
Bellevue MRN	
Elmhurst MRN	
Gouverneur MRN	
Last Name<br>* must provide value	
Middle Name	
First Name<br>* must provide value	
Date of birth<br>* must provide value	Today M-D-Y
SSN	
Last 4 SSN	
Gender	Female Male Other Unknown<br>reset
Race	Asian<br>Black or African American<br>Native Hawaiian/Pacific Islander<br>Unknown<br>White<br>Other<br>reset
Ethnicity	Hispanic<br>Non-Hispanic<br>Unknown<br>reset
Date of death (if applicable) (clinical source)	Today M-D-Y
Date of death (if applicable) (National Death index (NDI) source)	Today M-D-Y
Research consent form signed	Yes No Pending<br>reset

**Cancer Type (cancer 1)** * must provide value	Other
**Other cancer type (cancer 1)**	
**Source of data (cancer 1)** ☐ Clinical records ☐ New York tumor registry ☐ Connecticut tumor registry ☐ New Jersey tumor registry ☐ Florida tumor registry	
**ICD.9 Description/Code (cancer 1)**	
**ICD.10 Description/Code (cancer 1)**	
**NIOSH certified (cancer 1)**	○ Yes ○ No ○ Pending reset
**Laterality (cancer 1)**	○ Left ○ Right ○ Bilateral ○ Not applicable ○ Unknown reset
**Date of diagnostic procedure (cancer 1)**	Today M-D-Y
**Age at diagnosis (cancer 1)**	View equation
**Latency period (cancer 1) (months)**	View equation
**Place of initial pathology report (cancer 1)**	
**Multiplicity (# of tumors at primary site) (cancer 1)**	○ 1 ○ 2 ○ 3 ○ 4+ ○ Not applicable reset
**Tumor margins (cancer 1)**	○ Positive ○ Negative ○ Close margins ○ Unknown ○ Not applicable reset
**Grade (cancer 1)**	
**Histology (ICD-O-3) (cancer 1)**	
**pT-Primary Tumor (cancer 1)**	
**Tumor size (cm) (cancer 1)**	
**pN-Regional Lymph Nodes (cancer 1)**	
**pM-Distant metastasis (cancer 1)**	
**Stage (cancer 1)**	

**Biomarker (1) name (cancer 1)**	
**Biomarker (1) date (cancer 1)**	Today M-D-Y
**Biomarker (1) specimen type (cancer 1)**	
**Tumor marker (1) result interpretation (cancer 1)**	
**Tumor marker (1) result (cancer 1)**	
**Tumor biomarker (1) result unit (cancer 1)**	
**Biomarker (1) lab name (cancer 1)**	
**Is there another biomarker?**	○ Yes ○ No reset
**Comments (cancer 1)**	Expand
**Does patient have second cancer? (2)**	○ Yes ○ No reset
**Form Status**	
**Complete?**	Incomplete
**Lock this instrument?** If locked, no user will be able to modify this instrument for this record until someone with Instrument Level Lock/Unlock privileges unlocks it.	☐ Lock
	Save & Exit Form
